# Glymphatic solute transport does not require bulk flow

**DOI:** 10.1038/srep38635

**Published:** 2016-12-08

**Authors:** Mahdi Asgari, Diane de Zélicourt, Vartan Kurtcuoglu

**Affiliations:** 1 The Interface Group, Institute of Physiology, University of Zurich, Zurich, Switzerland; 2 Neuroscience Center Zurich, University of Zurich, Zurich, Switzerland; 3 Zurich Center for Integrative Human Physiology, University of Zurich, Zurich, Switzerland

## Abstract

Observations of fast transport of fluorescent tracers in mouse brains have led to the hypothesis of bulk water flow directed from arterial to venous paravascular spaces (PVS) through the cortical interstitium. At the same time, there is evidence for interstitial solute transport by diffusion rather than by directed bulk fluid motion. It has been shown that the two views may be consolidated by intracellular water flow through astrocyte networks combined with mainly diffusive extracellular transport of solutes. This requires the presence of a driving force that has not been determined to date, but for which arterial pulsation has been suggested as the origin. Here we show that arterial pulsation caused by pulse wave propagation is an unlikely origin of this hypothetical driving force. However, we further show that such pulsation may still lead to fast para-arterial solute transport through dispersion, that is, through the combined effect of local mixing and diffusion in the para-arterial space.

Recent observations of fast paravascular transport of exogenous fluorescent tracers in the mouse cortex have led to debates about the existence of bulk fluid flow through the brain’s extracellular space (ECS)^1^. Such bulk flow, directed from arterial to venous paravascular spaces, has been suggested to play an important role in cerebral metabolite clearance, with more efficient removal of solutes during sleep, and reduced clearance in aquaporin-4 (AQP4) deficient animals^2^,^3^.

Since cerebral water flow cannot be observed directly *in vivo* with sufficiently high resolution, the spatial and temporal variation of tracers is used as a surrogate marker for fluid motion. If a given tracer distributes more quickly than predicted by its diffusion coefficient, and if the distribution pattern is non-uniform, directed transport of the tracer by bulk water flow is usually suspected^4^. However, similar distribution patterns can also be caused by dispersion without directional bulk flow. Here, dispersion refers to transport of solutes by the combined effect of diffusion and macroscopic fluid motion with zero mean (see Fig. 1). For instance, cyclic deformation of the human brain induced by cardiovascular and respiratory action induces ventricular cerebrospinal fluid (CSF) motion with close to zero net flow that leads to substantial dispersion^5^. Similarly, dispersion occurs in the spinal canal through oscillatory CSF motion^6^.

In the absence of tools for measuring water flow directly, it is not straight forward to determine whether fast solute transport is the result of bulk flow or dispersion. Computational modeling can be used to assess the plausibility of either mode. We have shown previously that under the assumption of a para-arterial to para-venous driving force, the observed fast transport of solutes can be explained by bulk water motion, provided that there is substantial intracellular water flow through astrocytes^7^. It has been suggested, but not demonstrated, that arterial pulsation could generate the required driving force^8^,^9^.

Here we show using a new set of computational models that arterial pulsations caused by pulse wave propagation are unlikely to generate such driving force. Much rather than being the origin of bulk flow, we demonstrate that arterial pulsation may lead to fast paravascular solute transport by dispersion.

## Methods

We designed two distinct computational models: The first one was used to assess the impact of arterial pulsation alone on water and solute dynamics in the para-arterial space. With the second model we explored solute transport from the para-arterial to the para-venous space through the cortical interstitium. All model parameters are listed in Table 1.

### Model of water and solute dynamics in the para-arterial space

**Figure 2:**
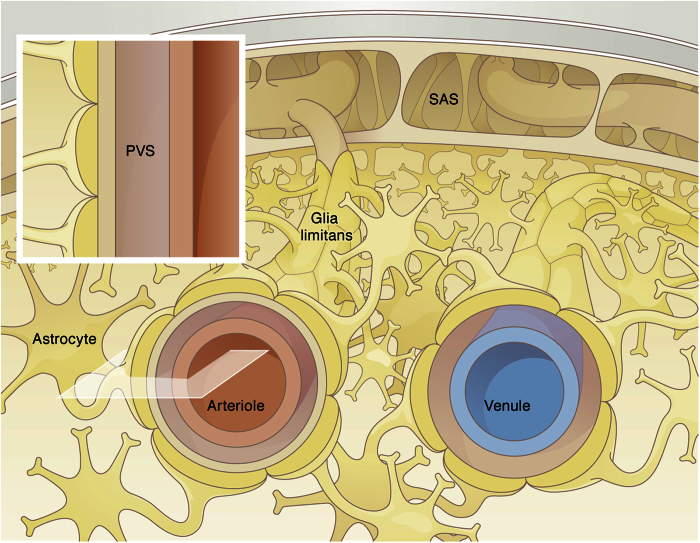
Schematic of cerebral arterial and venous paravascular spaces. The arterial PVS extends from the subarachnoid space (SAS) and follows the penetrating vessel into the tissue. This space is restricted on the one side by the vascular wall (endothelial and smooth muscle cells) and on the other side by the glia limitans. Glial endfeet processes almost completely cover the PVS of the larger vessels. The glia limitans of the arterial PVS is attached to the pia matter that extends from the SAS into the parenchyma. The inset shows the section of the arterial PVS retained for the axisymmetric computational model domain.

We consider a three-dimensional axisymmetric channel with a pulsating boundary as a simplified representation of a bifurcation free segment of cortical para-arterial space. A two-dimensional illustration of the correspondence between the channel – henceforth referred to as the model domain – and the represented paravascular space segment is shown in Fig. 2.

The domain length corresponds to the characteristic bifurcation free length of a penetrating arteriole in the mouse cerebral cortex. The total length of a cortical penetrating arteriole is approximately 500 μm 10 (Table 1), but the longest bifurcation free segment is clearly shorter^10^,^11^. According to Yoshihara *et al*.^11^ and private communications with the senior author of that study, Dr. Kazuto Masamoto, arterioles in the diameter range of 24.2 ± 2.4 μm have an average segment length of 234 μm. To account for variability and to probe the influence of segment length on the reported results, we consider a range of domain lengths from 150 to 250 μm.

**Figure 3:**
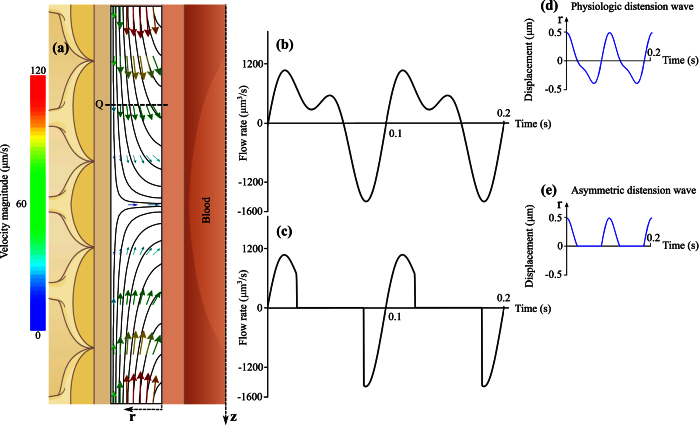
Fluid motion induced by vascular pulsation in a representative segment of arterial PVS of 150 μm length and 10 μm width. (**a**) Illustration of instantaneous flow field in the segment at the beginning of the pulsation cycle as shown in panel d. Depicted are streamlines and velocity vectors color coded according to velocity magnitude. While the highest speed shown in this figure is 120 μm/s, the maximum value reached throughout the cycle is 276 μm/s. (**b**) Flow rate induced by physiologic arterial pulsation measured at plane Q indicated in panel a. While instantaneous flow rates of up to 1590 μm^3^/s are observed, the net flow rate averaged over one cycle is only 0.372 μm^3^/s. (**c**) Flow rate induced by an artificial, asymmetric distension wave as shown in panel e. This wave was derived from the physiologic one shown in panel d by setting all negative displacements to zero. The goal was to obtain the highest possible flow rate without changing the wave amplitude, frequency and length. The net flow rate measured at plane Q is 1.179 μm^3^/s, thus still very small.

The model domain is treated as a CSF-filled porous medium. The domain outer boundary, bordered by the glia limitans, is considered stationary. The inner PVS boundary, bordered by the arterial wall, deforms transiently with the passage of pulse waves. The arterial wall motion is derived by normalizing the arterial distension wave function from Fujikura *et al*.^12^ with respect to the diameter of the considered cortical arteriole and applied to the inner boundary of the model domain (Fig. 3d). To characterize the sensitivity of our findings to the shape of the pulse wave, an alternative, asymmetric motion is derived from this first one by only considering the positive boundary displacements (Fig. 3e), thereby providing the most favorable condition for pulse wave-induced net flow in the PVS. The hydraulic resistance of the glia limitans layer is three to four orders of magnitude higher than that of the PVS^7^. Accordingly, the outer boundary of the axisymmetric domain is considered impermeable. Zero slip and zero solute flux are imposed on both the inner and outer boundaries. Zero velocity gradient, constant zero pressure and constant solute concentration are imposed at the axial boundaries.

The time-dependent equations governing fluid motion and solute transport, namely modified Navier-Stokes with Darcy’s law for the porous medium, continuity and advection-diffusion equations, are solved numerically using the open source finite volume code OpenFOAM^13^:


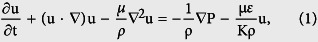



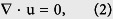



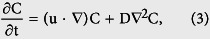


where the unknowns u, P and C are fluid velocity, pressure, and solute concentration, respectively. The parameters μ and ρ are, respectively, the dynamic viscosity and density of the cerebrospinal fluid, ε and K the porosity and permeability of the paravascular space, and D the diffusion coefficient of the respective solute. The parameter values are reported in Table 1.

Equations (1) to (3) are discretized using an implicit Euler scheme for the temporal derivatives and central differencing for the first and second order spatial derivatives. All calculations were conducted with a time step size of 5 · 10^−5^ s and spatial resolution of 0.25 μ*m* × 0.5 μ*m* along the axial and radial directions, respectively. Grid and time step independence was confirmed.

### Dispersion coefficient determination

We postulate that the effect of arterial pulsation on solute transport in a model domain of length L can be approximated by the dispersion equation


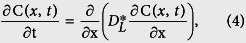


where x is the spatial coordinate in axial direction and 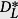 is the dispersion coefficient in a segment of length L. This equation is structurally equivalent to the well-known diffusion equation in one dimension^14^. To evaluate how closely the dispersion equation captures transport in the arterial PVS, we consider the analytical solution of equation (4) in a semi-infinite domain,


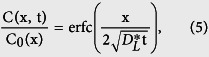


where C_0_ is the initial concentration. For a finite domain, this approximation is valid as long as the penetration Fourier number for the domain length remains small^15^. The value of 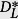 is determined by fitting Equation (5) to the results of the axisymmetric simulations at t = 10 s. Using other time points that still fulfill the above Fourier criterion results in the same value of 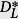 (maximum deviation of 1%).

**Figure 4:**
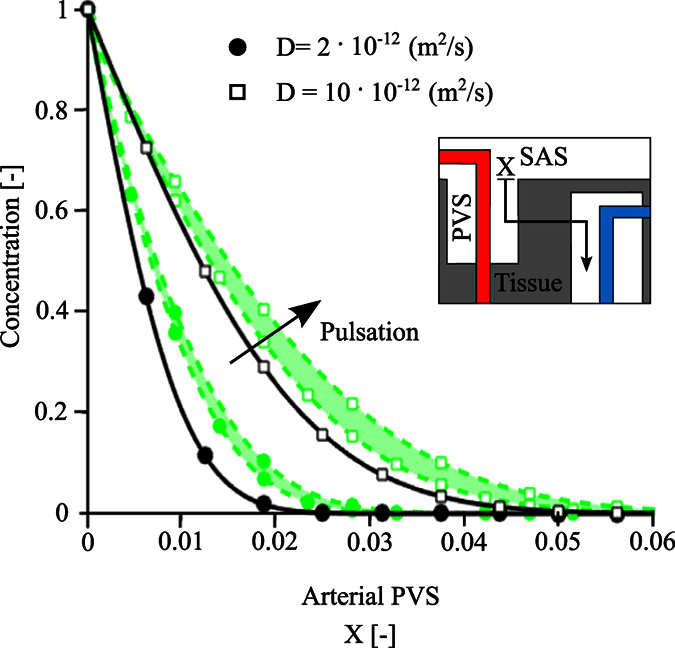
Transport of solutes in the arterial PVS in the presence (green lines and symbols) and absence (black lines and symbols) of arterial pulsation. Solutes with diffusion coefficients of 2 · and 10 · 10^−12^ m^2^/s are considered (circles and squares, respectively). Symbols show the concentration profiles obtained from the 3D axisymmetric simulations 10 seconds after the entrance of solutes from the arterial PVS-SAS interface at X = 0. Lines illustrate the best fit curves obtained for the diffusion (continuous black lines) and dispersion (dotted green lines) cases. As dispersion effects depend on domain length, dispersion results are reported for a bifurcation-free arteriole segment length spanning 150 μm (lower bound of the shaded area) to 250 μm (upper bound). The effect of arterial pulsation can be approximated by the analytical solution of the dispersion equation (equation (5)) without having to account for pulsation explicitly. Corresponding dispersion curves (dotted lines) obtained using dispersion coefficients of 3.5 · and 4.2 · 10^−12^ m^2^/s (lower and upper green circles) and 12.7 · and 17.0 · 10^−12^ m^2^/s (lower and upper green squares) accurately reproduce the effect of pulsation (coefficient of determination: R^2^ > 0.99).

As shown in Fig. 4, the dispersion equation captures the transport characteristics very well (coefficient of determination: R^2^ > 0.99).

### Model of solute transport from arterial to venous paravascular space

**Figure 5:**
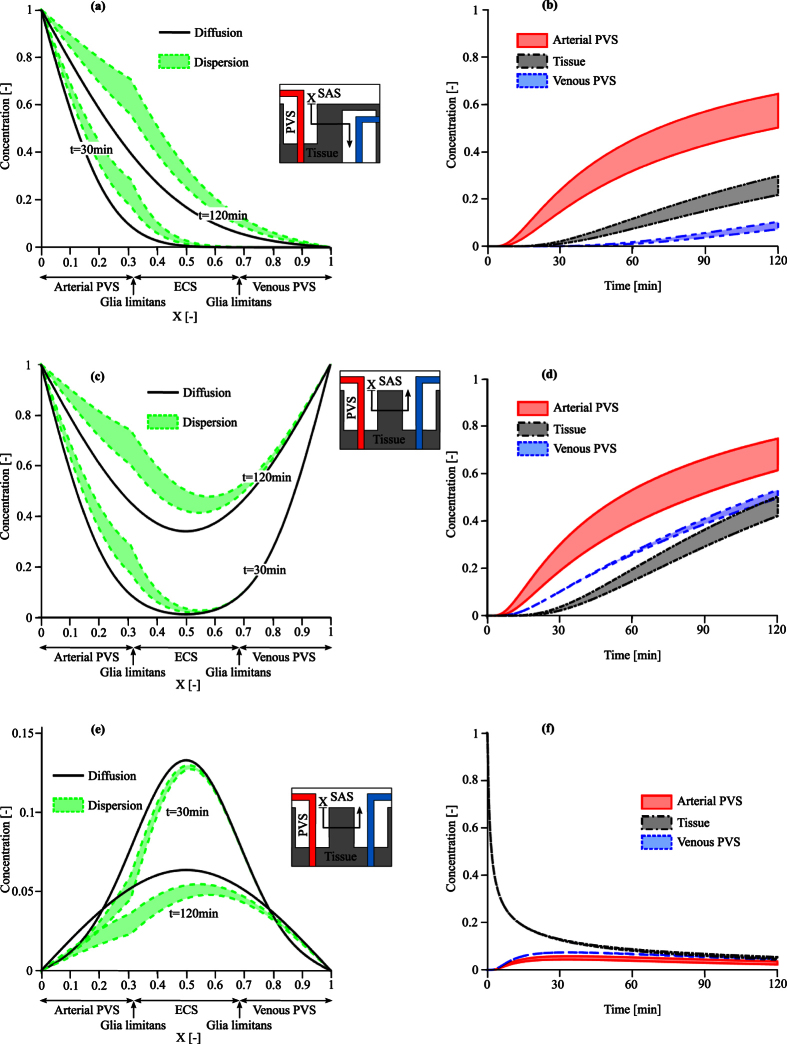
Solute transport in the brain for particles of 14 nm size (Dextran 70) with a diffusion coefficient of 6 · 10^−12^ m^2^/s under three different injection scenarios: (**a**,**b**) Case A, cisternal injection with a concentration gradient between arterial and venous PVS interfaces, (**c**,**d**) Case B, cisternal injection with no concentration gradient, and (**e**,**f**) Case C, interstitial injection. Left column: comparison of the concentration profiles at 30 and 120 mins with dispersion and pure diffusion. Dispersion results (colored bands) represent a range of plausible dispersion coefficients as reported in Table 2. Right column: Time evolution of the solute concentration due to dispersion in the arterial PVS (at X = 0.3), ECS (X = 0.5), and venous PVS (X = 0.7).

**Figure 6:**
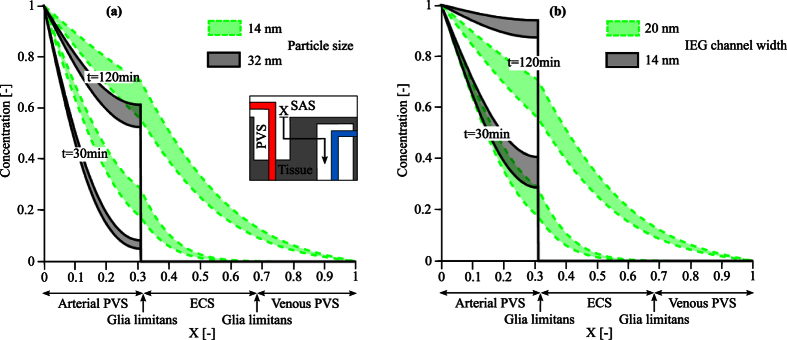
Spatial and temporal distribution of the solute concentrations in the brain (**a**) for two different particle sizes, 14 and 32 nm, respectively, assuming an IEG width of 20 nm (**b**) and for the 14 nm particle under the effect of IEG width reduction from 20 nm to 14 nm. Results represent a range of plausible dispersion coefficients as reported in Table 2. In both cases, the particles enter the arterial PVS from the arterial PVS-SAS interface. While in the normal glia limitans morphology (**a**) the larger particles (32 nm in size) are already trapped by this layer and cannot pass into the parenchyma, reduction of IEG width (**b**) also inhibits passage of the smaller solute.

In the second model, we consider a one-dimensional representation of the solute transport between arterial and venous PVS. Our domain consists of a 250 μm long segment of para-arterial space, associated 1 μm thick segment of glia limitans, 300 μm of cerebral cortical tissue separating the artery-vein pair, and again a 1 μm thick segment of glia limitans and 250 μm of para-venous space. All distances are normalized by the total domain length, where X = 0 and 1 correspond to the points where the para-arterial and para-venous spaces, respectively, are exposed to the subarachnoid space (see X-axes of Figs 5 and 6).

Based on the observations that 1) arterial pulsations yield negligible bulk flow in the physiological regime (Fig. 3) and 2) the dispersion equation provides a very good approximation of solute transport in the arterial PVS (Fig. 4), we use equation (4) to describe solute transport inside our one-dimensional domain. Across the glia limitans and in the cortical ECS, diffusion dominates due to the high hydraulic resistance of the interstitial pathways^7^,^14^. In the venous PVS, pulsations propagating in axial direction are expected to be small, since arterial pulse waves are damped in the capillary bed. Accordingly, we assume that the dispersion coefficient reduces to the effective diffusion coefficient of solutes across the glia limitans and in the cortical ECS and para-venous spaces. In the para-arterial space, we use the dispersion coefficient determined from the axisymmetric simulations.

Large brain metabolites (such as amyloid beta) or tracers (such as Dextran 70) are comparable in size to the characteristic width of the ECS and gaps between astrocyte endfeet (inter-endfeet gaps or IEG)^14^ through which they have to pass on their way from PVS to ECS. As a consequence, the effective diffusion coefficient of these larger solutes in their passage through IEG and ECS is lower than that for free diffusion in the same fluid. The relation between effective and free diffusion coefficients is given according to Deen *et al*.^16^ by


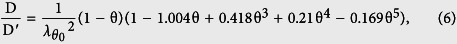


where D is the effective diffusion coefficient, D′ is the corresponding free diffusion coefficient, θ is the size ratio of the solute to the relevant channel or ECS dimension and 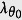 is the path tortuosity determined for a vanishingly small molecule. This equation is used to determine changes in the effective diffusion coefficient of solutes when dimensions of IEG or ECS are altered, e.g. due to the effects of AQP4 deletion. The equation for solute transport is solved using finite difference discretization in Matlab with a forward Euler time stepping scheme, second order central differences for the spatial second derivatives, a time step size of 0.01 s and a spatial resolution of 1 μm.

Dirichlet boundary conditions for concentrations are imposed on the proximal arterial and distal venous PVS interfaces. The exact anatomical configuration of the venous and arterial PVS is still a matter of debate and investigation^17^. While artery-vein pairs have been depicted to penetrate the cortex from the cranial SAS in close proximity to each other^18^, they have, in contrast, also been suggested to have distinctively different starting and end points, with artery and vein penetrating the cortex from different regions of the SAS or only the artery reaching from the SAS into the cortex^19^.

We take into account these two anatomical configurations separately to study three distinct cases of solute transport concisely illustrated in the insets of Fig. 5. Case A represents the second configuration with the vein returning to the SAS at a distinctively different location than where the artery penetrates the cortex. Consequently, there may be different solute concentrations at the arterial PVS inlet and venous PVS outlet. For Cases B and C, the first configuration is used, where the SAS entry and exit locations of the artery and vein PVS are next to each other, and inlet and outlet solute concentrations are thus assumed to be equal at all times.

Cases A and B consider the transport of solutes originating in the cisterna magna, e.g. due to tracer injection. While the solute concentrations at the arterial and venous PVS-SAS interface are equal in Case B, there are different concentrations in the arterial and venous interfaces in Case A due to the mentioned difference in vascular configuration. In both cases, the initial tracer concentration is set to 0 throughout the domain and to 1 at the arterial PVS-SAS interface. Concentration at the venous PVS-SAS interface is set to 0 and 1 in Cases A and B, respectively.

Case C considers the transport of solutes originating in the parenchymal interstitial space. To mimic interstitial tracer injection, the initial solute concentration is set as a rectangular function with the value of 1 in a 50 μm wide section at the center of the considered parenchymal segment and 0 elsewhere. Given the difference in volume between PVS and SAS, solute concentration in the former is assumed to not affect the latter, and the solute concentration is maintained at zero at both arterial and venous PVS boundaries.

## Results

### Impact of arterial pulsation on water dynamics in the PVS

We interrogated the first model to evaluate whether arterial pulsation may induce bulk flow in the PVS. To this end, we prescribed the axially propagating distension wave shown in Fig. 3d to the inner boundary of the model domain, and calculated the resulting fluid flow. A representation of the flow field by streamlines and velocity vectors is shown in Fig. 3a. While rather high instantaneous axial flow rates of up to 1590 μm^3^/s are reached (Fig. 3b), their temporal average over a cardiac cycle is three orders of magnitude lower. This is because the ratio of axial PVS length to the wavelength of the distension wave is very small, approximately 1.5 · 10^−3^. Consequently, the arterial wall motion is perceived on a local scale as a uniform radial displacement rather than a peristaltic motion driving CSF forward, and the resulting bulk CSF flow rate in the PVS is 0.372 μm^3^/s. This flow rate is insensitive to variations of the arterial pulsation characteristics within a reasonable range. A change in the distension wave characteristic shape (to make it more asymmetric and thus more likely to produce net flow, Fig. 3c and e) only increases the bulk flow rate to 1.179 μm^3^/s. Even when considering the maximum conceivable domain length of 500 μm, which corresponds to the total characteristic length of a penetrating arteriole, considering the lower bound of the wave propagation velocity (0.1 m/s), the upper bound of the mouse heart rate (660 bpm) and high displacement amplitude of 1 μm, we obtain a net bulk flow rate of 3.56 μm^3^/s, which is still negligibly small.

### Solute transport in the arterial PVS

We used again the first model to compare the speed of solute transport through the arterial PVS by pure diffusion, pure advection (bulk water flow) and dispersion. We considered solutes with cerebral diffusion coefficients in the range of 2 · 10^−12^ to 10 · 10^−12^ m^2^/s, which corresponds to larger brain metabolites and to tracers used to study paravascular flow^2^,^14^.

Table 2 lists the distribution length of these solutes determined by first setting the normalized solute concentration to 0 inside the model domain and to 1 at the interface with the SAS, and then measuring after 10 seconds the distance at which the solute concentration in the PVS has reached 50% of the initial SAS value. In the context of pure advection, distribution lengths after 10 seconds are independent of tracer size and two orders of magnitude smaller than with diffusion, reflecting the slow mean bulk flow reported above. In contrast, dispersion yields distribution lengths that are 16% to 50% longer than those obtained with diffusion alone. The effect of dispersion is more pronounced for larger solutes with smaller diffusion coefficients.

Figure 4 illustrates the concentration profiles of the two tracers (D =  2· and 10 · 10^−12^ m^2^/s) at 10 seconds in the context of dispersion and pure diffusion. This figure re-emphasizes that solutes spread farther under the effect of dispersion than with diffusion alone. Equation (5) (dashed lines) provides an excellent approximation of the model results (symbols; coefficient of determination: R^2^ > 0.99). The dispersion coefficient obtained through equation (5) depends on the solute considered, but also on the periodic flow induced by the arterial distension wave. The latter is a function of PVS segment length. Calculated dispersion coefficients are given in Table 2, and used in the second model to extend our study of solute transport to the brain tissue and venous PVS.

### Solute transport from para-arterial to the para-venous space

We interrogated the second model to assess whether dispersion in the arterial PVS may enhance overall cortical solute transport despite the absence of bulk flow. To that end, we investigated two different injection scenarios: into the cisterna magna (Cases A and B), and directly into the parenchymal interstitium (Case C).

Dispersion caused by arterial pulsation leads, as expected, to faster solute transport in the para-arterial space than pure diffusion (Fig. 5a). As a result, solutes reach the interface between PVS and tissue faster, which in turn enhances solute transport through the glia limitans and ECS. The higher resistance of glia limitans and ECS to solute passage breaks the smoothness of the graph at the location of the glia limitans. Figure 5b shows the corresponding time evolution of the solute concentration in the arterial PVS, tissue and venous PVS. The directionality of the solute transport from arterial PVS to tissue and then venous PVS is reflected by the time shift between the different curves.

The difference in para-arterial and para-venous transport is best seen in Cases B and C (Fig. 5c–f), where identical concentrations are imposed on the arterial and venous interfaces. When considering pure diffusion, the symmetry of the boundary conditions results in symmetric concentration profiles (Fig. 5c and e). For cisternal injection, dispersion results in faster penetration along the arterial PVS (Fig. 5c and d). The slower diffusion in the tissue leads to a large concentration gradient between arterial PVS and tissue for the entire simulated period of 120 minutes. In contrast, solute concentrations in the venous PVS and tissue remain of the same order (Fig. 5d). For interstitial injection, dispersion results in faster solute clearance in the arterial PVS (Fig. 5e) and longer tracer residence times in the venous PVS (Fig. 5f).

### Impact of glia limitans morphology on solute transport

The glia limitans plays a critical role in the system considered. As suggested by *in vivo* observations in AQP4 deficient animals, it may modulate solute transport and fluid fluxes between parenchyma and PVS^2^.

Under normal conditions, the inter-endfeet gap between adjacent astrocytes allows for the passage of solutes of up to 20 nm hydraulic diameter, while blocking larger ones (see Fig. 6a). Amiry-Moghaddam *et al*.^20^ and Manley *et al*.^21^ report that AQP4 deletion or depolarization also induce changes in astrocyte endfoot morphology, possibly due to changes in the balance of osmotic forces in intra- and extracellular spaces. Should these morphological changes yield an increase in astrocyte endfeet volume or coverage area, they may concurrently reduce IEG width and thereby the permeability of the glia limitans. To assess whether such hypothetical changes could yield significant changes in the overall rates and patterns of tracer transport, we performed a supplementary set of calculations with reduced permeability of the glia limitans corresponding to a 30% reduction of IEG width from 20 nm to 14 nm. As shown in Fig. 6b, such reduction in permeability leads to a remarkable reduction of solute transport through the ECS, while transport through PVS stays high. This is in line with observations in AQP4 deficient animals^2^.

## Discussion

Cerebral water dynamics cannot be measured *in vivo* with sufficient resolution to support or challenge the hypothesis of cortical paravascular bulk flow. We designed two computational models to fill this gap. The first one was created to test whether arterial pulsations, which have been suggested as possible driving mechanism for bulk flow, can convey solutes at rates demonstrated by tracer studies^2^,^22^,^23^,^24^. The second model was designed to test whether dispersion induced by arterial pulsation may be responsible for the observed fast para-arterial solute transport.

The possibility of pulsation-driven bulk flow through the arterial PVS has been investigated before by computational^25^ and analytical modeling^26^. The analytical solution of Wang *et al*.^26^ yields unrealistically high mean PVS velocities on the order of cm/s, and is independent of the distension wavelength. In contrast, Bilston and co-workers came to the conclusion that bulk flow is possible for short distension wavelengths of 20 to 300 μm and decreases as the wavelength increases. However, they did not extend their study to physiologic wavelengths, which are three to four orders of magnitude longer^27^. Our results show that under physiologic conditions, arterial pulsations alone are unlikely to produce notable bulk flow. The absence of bulk flow in the PVS does not imply absence of substantial local fluid motion. Indeed, our results show that arterial pulsation-induced local fluid motion leads to fast solute transport by dispersion even though there is close to zero directed bulk flow. This is in line with observations of reduced solute transport in the PVS after aortic occlusion^9^ or internal carotid artery ligation^8^, which reduce cerebral arterial pulsation and may thereby reduce dispersion.

The hypothesis of paravascular bulk flow directed from the arterial to the venous side has not yet been reconciled with reports of solute movement in the opposite direction: Both tracers injected into the parenchyma^22^,^23^ and large endogenous proteins^28^ have been shown to spread diffusely through the parenchyma and then drain out along the arterial wall towards the SAS. Arterial paravascular solute transport by dispersion can account for this behavior: Depending on the origin of the solute, fast transport can occur towards (Fig. 5e) or away (Fig. 5a,c) from the SAS. Since dispersion is caused by arterial wall pulsation, transport is faster in the arterial than in the venous PVS. This results in faster tracer penetration along the arterial PVS after cisternal injection (Fig. 5d) or faster clearance of the para-arterial space and longer tracer residence time in the venous PVS after interstitial injection (Fig. 5f), both of which are in line with *in vivo* observations^2^. Another indication for pulsation-mediated para-arterial transport is the increased deposition of amyloid-beta in the arterial basement membrane of old brains^28^. As arteries stiffen with age, their distension amplitude decreases, thereby reducing dispersion in the arterial PVS and increasing protein residence time, which may lead to increased deposition. The observation that deposition occurs on the arterial rather than venous side (where transport is slower to begin with) might be explained by higher affinity of amyloid beta to structures in the arterial wall.

The main pillar of the bulk flow hypothesis is that in mice lacking AQP4, tracer transport in the ECS is substantially reduced compared to their wild type counterparts. However, the corresponding experiments also show that transport in the PVS is affected much less^2^. If there is bulk flow from the para-arterial space through the ECS into the venous PVS, the question arises as to how flow in one segment of this chain (ECS) can be reduced substantially while flow in another segment (para-arterial space) is affected only marginally. Indeed, we have shown previously that reduced flow through the ECS necessitates reduced flow in the PVS to be in line with the bulk flow hypothesis^7^.

While the alternative hypothesis of paravascular solute transport by dispersion is consistent with observations of limited change in tracer spread in the para-arterial space in AQP4-deficient animals, it does pose challenges with respect to reduced extracellular transport: under the assumption that the deletion of AQP4 has no other effect than increasing trans-membrane resistance to water flux, our model does not predict any reduction in the speed of tracer spread in the ECS because diffusion is dominant in that region^7^,^14^,^29^.

Since neither the bulk flow nor the dispersion hypothesis can fully account for the transport behavior in absence of AQP4, it is necessary to question the premise of no secondary effects caused by the deletion of these water channels. As a matter of fact, there are several reports of changes in parenchymal diffusion and ECS volume fraction caused by AQP4 deletion^30^,^31^. However, these studies show increased extracellular diffusion fluxes and ECS volume fraction in animals lacking AQP4, conflicting with studies showing markedly reduced rates of tracer penetration into the parenchyma^2^. This conflict may be resolved by considering how the corresponding studies were carried out: while local ECS diffusion was determined with brain surface photobleaching^30^ and TMA^+^ iontophoresis^31^, tracer transport was recorded on a larger scale^2^, where the passage of solutes between ECS and PVS through the glia limitans becomes relevant.

The relevant structures for such solute transfer are the astrocyte inter-endfeet gaps. Beyond global changes in ECS volume fraction, there are indications that the endfoot morphology may also be affected by AQP4 deletion or depolarization^20^,^21^, possibly due to the changed balance of osmotic forces and intra-/extra-cellular volume regulation^32^. These morphological changes may reduce the permeability of the glia limitans. The remarkable reduction of ECS solute transport shown in Fig. 6 indicates that reasonable morphological changes may limit the transport of small solutes past the glia limitans, just as this structure limits the transport of larger solutes such as FITC-d2000 under normal conditions^2^.

As any model, the two computational representations used in this study have their limitations. First and foremost, we have simplified the cerebral anatomy substantially to three dimensional axisymmetric and one dimensional representations, respectively, and considered the PVS as a homogenous porous medium. It is principally possible that anisotropies in the PVS may act as one-way valves, enabling bulk flow together with arterial pulsation. However, such valves, while introduced as a hypothetical concept^33^, have not been found to date. Furthermore, the still debated matter of the venous drainage site limits the definition of exact boundary conditions. To address that difficulty, we characterized solute transport under two extreme scenarios where the solute concentration on the distal venous PVS interface is either null or equal to the proximal arterial one.

Next to anatomical approximations, the issue of uncertain parameters is a recurring challenge in biophysical modeling. We have dealt with it by performing sensitivity analyses to ensure that the conclusions of our study hold within a reasonable parameter range. In particular, the reported dispersion coefficients are independent of the PVS hydraulic conductivity, but depend on instantaneous flow rates and thereby on PVS segment geometry as well as wave characteristics. Importantly, dispersion increases with increasing segment length. We have accounted for this by reporting results for a range of bifurcation free segment lengths of 150–250 μm. While there are both shorter and longer segments in the mouse cerebral cortex, their average length in the diameter range considered here is 234 μm. We chose the upper value of the segment length range purposefully close to this average in order not to overstate the dispersion effect on solute transport in the PVS.

While very limited data exist on the exact pulse waveform in mouse cortical arterioles, our sensitivity analysis indicates that even an extreme asymmetric arterial distension waveform does not produce notable bulk flow. Other waveforms not caused by arterial pulse wave propagation but due to, for example, local cyclic vascular contraction could principally cause bulk flow^34^, provided that their wavelengths are orders of magnitude smaller than that of the arterial pulse wave^25^. Otherwise, they would simply increase dispersion. No such waves have been identified to date.

Finally, the modeled effects of possible glia limitans permeability reduction due to AQP4 knock-out need to be viewed as exploratory until corresponding changes to astrocyte endfeet have been shown conclusively *in vivo*. In general, a better characterization of differences in paravascular, interstitial, intracellular and cerebrospinal fluid pathways between wild type and AQP4 deficient mice is necessary to correctly interpret the results of tracer studies.

In summary, we have shown that arterial pulsations may lead to fast transport along the arterial PVS due to dispersion, but not due to bulk flow. This may reconcile a number of apparently conflicting experimental observations, notably transport in opposite directions in the PVS, i.e. either from the SAS to the parenchyma or the reverse, and faster transport in the arterial PVS compared to the venous one, which the net water flow assumption could not. While the existence of dispersion does not exclude the possibility of bulk flow, the latter would require a yet unidentified driving force as well as substantial intracellular flow through astrocyte networks^7^. Our study shows that arterial pulsation is unlikely to function as that driving force.

## Additional Information

**How to cite this article:** Asgari, M. *et al*. Glymphatic solute transport does not require bulk flow. *Sci. Rep.* **6**, 38635; doi: 10.1038/srep38635 (2016).

**Publisher's note:** Springer Nature remains neutral with regard to jurisdictional claims in published maps and institutional affiliations.

## Figures and Tables

**Figure 1:**
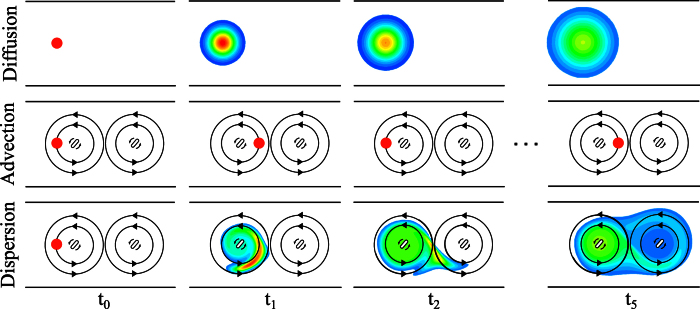
Illustration of the differences between solute transport by diffusion, advection and dispersion in the absence of net flow. In this hypothetical setup, which is not meant to represent paravascular transport, a drop of solute (red circle) is injected into a fluid filled channel at time t_0_ (left column). In the top row, the fluid is still and the solute may solely be transported by diffusion, whereas in the two other conditions (advection, middle row, and dispersion, bottom row), two stir bars rotating counterclockwise induce a continuous fluid motion with zero net flow. The temporal evolution of the solute concentration (red: high, blue: low concentration, white: no solute) is illustrated in the following columns at equally spaced time points, **t**_**i***** ***= **0…5**_. Pure diffusion leads to slow solute transport. Advection by itself moves the solute much faster, but, in absence of any diffusion, confines the drop to the influence region of the first stir bar. In contrast, the combined effects of advection and diffusion, i.e. dispersion, result in fast solute transport from left to right, even though there is no (time-averaged) net flow of the liquid.

**Table 1:** Model parameters.

Parameter	Value	Unit	Reference
**3D Axisymmetric model of water and solute dynamics in arterial PVS**			
Arterial wall distension wave			
Wave amplitude	0.5	μm	8
Wave propagation velocity	1	m/s	26
Wave frequency	10	1/s	35
Wavelength	0.1	m	
Dimensions			
Cortical arteriole diameter	23	μm	8
PVS model domain length (L)	150–250	μm	11
PVS model domain width (W)	10	μm	2
PVS domain properties			
PVS hydraulic permeability (K)	2.10^−12^	m^4^/*N* · *s*	26
PVS porosity (*ε*)	0.2	—	26
Cerebrospinal fluid properties			
Density (*ρ*)	1000	kg/m^3^	36
Dynamic viscosity (μ)	0.001	Pa · s	37
**1D model of solute transport in PVS and ECS**			
Half-length of the paravascular space of a penetrating vessel	250	μm	10
Thickness of the glia limitans	1	μm	38
Characteristic distance between arteriole and venule PVS	300	μm	39
IEG width	20	nm	2, 40
Characteristic ECS channel width	20	nm	41
ECS volume fraction (*α*)	0.2	—	14

**Table 2:** Dispersion coefficients and distribution lengths of the three solutes considered in the study.

Diffusion coefficient (m^2^/s)	Dispersion coefficient (m^2^/s)		Distribution length (μm)			
	L = 150 μm	L = 250 μm	Diffusion	Advection	Dispersion, L = 150 μm	Dispersion, L = 250 μm
2.10^−12^	3.5∙10^−12^	4.2∙10^−12^	4	0.24	5.4	6.0
6.10^−12^	8.1∙10^−12^	10.7∙10^−12^	7	0.24	8.4	9.3
10.10^−12^	12.7∙10^−12^	17.0∙10^−12^	9	0.24	10.4	12.0

The dispersion coefficient values are reported for PVS segment lengths, L, of 150 and 250 μm. The distribution lengths are determined in the context of pure diffusion, pure advection and dispersion by first setting the normalized solute concentration to 0 in the arterial PVS and 1 in the SAS, and then measuring after 10 seconds the distance at which the solute concentration in the PVS has reached 50% of the initial SAS value.
